# LAPTM4B as a key regulator in the copper-induced endoplasmic reticulum–lysosome interplay disorder in duck liver and the protective role of baicalin

**DOI:** 10.1186/s40104-025-01255-0

**Published:** 2025-09-01

**Authors:** Hongyu Shang, Xueyan Dai, Jing Chen, Chenghong Xing, Xiaona Gao, Huabin Cao, Guoliang Hu, Haotang Li, Mingwen Hu, Fan Yang

**Affiliations:** Jiangxi Provincial Key Laboratory for Animal Health, Institute of Animal Population Health, College of Animal Science and Technology, Economic and Technological Development District, Jiangxi Agricultural University, No. 1101 Zhimin Avenue, Jiangxi, 330045 People’s Republic of China

**Keywords:** Baicalin, Copper, Endoplasmic reticulum-lysosomal crosstalk, Hepatotoxicity, LAPTM4B

## Abstract

**Background:**

Copper (Cu) is a pervasive environmental pollutant with significant hepatotoxic effects in animals. The endoplasmic reticulum (ER) interacts closely with lysosomes to maintain intracellular homeostasis. However, the role and mechanism of ER-lysosome crosstalk in Cu-induced liver injury in ducks remains unclear. To investigate this, we established both an in vivo model of Cu-exposed ducks and an in vitro model of duck hepatocytes, and added baicalin (Ba) to further explore its protective effects.

**Results:**

The results of this study demonstrated that exposure to Cu resulted in vacuolar degeneration and oxidative stress in duck hepatocytes, while ultrastructural observations revealed ER swelling and an increased number of autophagic lysosomes. Furthermore, Cu exposure significantly upregulated mRNA and protein levels related to ER stress, autophagy, and lysosomal membrane factors. It also markedly increased ER-lysosomal co-localization. Further experiments showed that knockdown of LAPTM4B significantly attenuated Cu-induced ER autophagy and reduced ER-lysosomal co-localization in hepatocytes. Molecular docking and molecular dynamics simulations confirmed that LAPTM4B has a stable binding site to Ba; in vitro experiments demonstrated that Ba could effectively alleviate Cu-induced ER-lysosome crosstalk in duck hepatocytes and reduce hepatocyte injury by targeting LAPTM4B; additionally, in vivo experiments showed that Ba significantly inhibits Cu-induced liver injury in ducks.

**Conclusions:**

In summary, the present study demonstrates that Cu exposure disrupts ER-lysosomal crosstalk in duck liver, leading to ER-lysosomal damage and subsequent hepatocyte injury. In contrast, Ba alleviates this injury by selectively targeting LAPTM4B, ultimately attenuating Cu-induced hepatotoxicity.

**Graphical Abstract:**

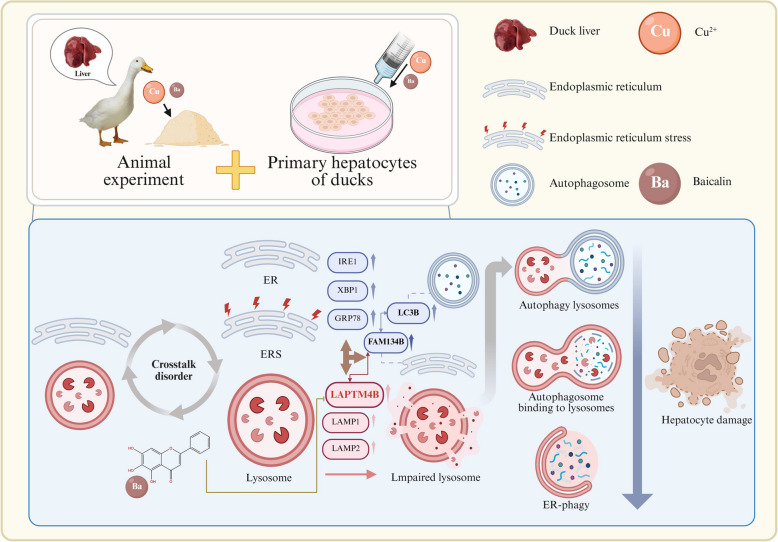

**Supplementary Information:**

The online version contains supplementary material available at 10.1186/s40104-025-01255-0.

## Introduction

Copper (Cu) is an essential trace element required for maintaining normal physiological functions in humans and is widely involved in various biological processes [1]. Although Cu is indispensable for life, excessive intake can lead to severe toxicity [2]. Study has shown that when Cu intake exceeds 2.09 mg/d, it may pose significant health risks [3]. With the rapid development of industrialization, the extensive use of Cu in electronics, metallurgy, and chemical industries has resulted in the release of large amounts of Cu pollutants into the environment [4]. In some areas, Cu concentrations in estuaries and coastal waters have been reported to range from 55.78 to 4,906.25 μmol/L, well above the World Health Organization (WHO) guideline limits for Cu in drinking water [5]. Additionally, Cu mining and smelting activities have led to a substantial rise in Cu levels in adjacent soils. In severely contaminated areas, Cu concentrations have exceeded 650 mg/kg, which is 44 times higher than the background Cu levels in the local soil [6, 7]. Through dietary intake and bioaccumulation, Cu primarily accumulates in the liver, leading to pathological changes in liver tissue [8]. Moreover, Cu induces the formation of reactive oxygen species (ROS) via the Fenton reaction, which subsequently initiates oxidative stress. This oxidative cascade leads to lipid peroxidation and protein oxidation, ultimately compromising cellular integrity and functionality [9]. Our previous study found that Cu exposure induces abnormal crosstalk between mitochondria-associated Endoplasmic reticulum (ER) membranes and autophagy, which sparked our interest in the role of organelle interactions in Cu-induced organ damage [10]. Recent studies have shown that there is a close interaction between the endoplasmic reticulum and lysosomes [11–13], particularly under stress conditions, and that ER autophagy (ER-phagy) depends on lysosomal function to degrade damaged ER components. Therefore, studying the crosstalk between the ER and lysosomes under Cu exposure is crucial for gaining a deeper understanding of the mechanisms underlying Cu toxicity.

The ER and lysosomes are two key organelles that maintain cellular homeostasis and regulate stress responses [14, 15]. The research on the association between the ER and lysosomes commenced in the mid-twentieth century. Initially, the two organelles were regarded as independent entities; however, subsequent studies have gradually revealed their functional connections. Specifically, the ER plays a pivotal role in lysosomal regulation through multiple mechanisms. First, it supplies lysosomes with hydrolytic enzymes (sorted via M6P [mannose-6-phosphate] labeling) and membrane lipid components (e.g., cholesterol and sphingomyelin). Second, it directly regulates lysosomal localization and metabolism through membrane contact sites (MCSs) (e.g., ORP1L-VAP complexes and NPC1-mediated cholesterol transport) [16, 17]. Furthermore, under stress conditions, the ER can trigger selective autophagy via the ER-phagy pathway. This process relies on receptors such as FAM134B to wrap the damaged ER into autophagosomes, which are ultimately degraded by lysosomes. Recent study emphasizing their dynamic crosstalk in orchestrating cell fate through integrated material exchange and signaling networks [18], while further cementing the ER’s role in autophagy-mediated clearance of dysfunctional components [19, 20]. Exacerbating intracellular homeostatic disturbances lies compromised ER degradation, a direct consequence of lysosomal functional impairment [21, 22]. Emerging evidence indicates that the lysosomal stress response mechanism involves coordinated actions of multiple regulatory factors, particularly during heavy metal-induced disruption of lysosomal membrane integrity [23]. Among these modulators, the lysosome-associated transmembrane protein (LAPTM) family has been recognized as a central regulatory hub. Notably, LAPTM4B stands out as a key family member characterized by its four transmembrane domains and lysosomal targeting signals [24]. Recent mechanistic investigations have revealed that LAPTM4B serves as a key modulator of autophagy, facilitating autophagosome-lysosome fusion via its regulatory effects on lysosomal membrane integrity and functional activity [25, 26]. Additionally, LAPTM4B is involved in lysosomal biogenesis and transport, thereby influencing the completion of autophagic flux [27, 28]. Although LAPTM4B has been shown to be essential for maintaining lysosomal stability and regulating autophagy, its involvement in the ER-lysosome crosstalk mechanism remains unclear.

Baicalin (Ba), a naturally occurring flavonoid compound predominantly isolated from *Scutellaria baicalensis* Georgi and other medicinal plants, demonstrates diverse pharmacological properties, most notably potent antioxidant and anti-inflammatory activities. In recent years, the protective role of Ba in heavy metal toxicity has gained attention [29]. Studies have shown that Ba can alleviate heavy metal-induced cellular damage by modulating chloride channels and suppressing excessive reactive oxygen species (ROS) production, while research also demonstrates a positive correlation between Ba presence and enhanced heavy metal excretion [30, 31]. Evidence suggests that Ba alleviates liver damage by stimulating hepatic CPT1 and NLRP3 activity [32, 33]. However, the target of Ba in Cu-induced hepatotoxicity and the underlying molecular mechanisms remain unexplored. Based on the above background, the present study aimed to elucidate the regulatory role of LAPTM4B in Cu-induced crosstalk between the hepatic ER and lysosomes and to explore the potential mechanism by which Ba attenuates Cu-induced hepatotoxicity through targeting LAPTM4B. This study elucidates novel molecular mechanisms underlying copper-induced hepatotoxicity and identifies potential therapeutic targets for mitigating and managing Cu-associated liver injury.

## Materials and methods

### Ethics statement

All animal experiments and procedures were approved by the Ethics Committee of the Jiangxi Agricultural University (Approval ID: JXAULL-2024-09-02).

### Animals and treatment

A total of 48 one-day-old Peking ducks were acclimated for 1 week under identical environmental conditions. They were then randomly assigned to four groups (all added through mixing): control group (the basic diet contains 10 mg/kg Cu), 400 mg/kg Cu group, 400 mg/kg Cu + 20 mg/kg Ba group, and 20 mg/kg Ba group. The doses of Cu and Ba were determined based on previous studies and incorporated into the basal diet [34, 35]. The experiment lasted 42 d, with 12 ducks in each group. At the end of the experiment, the ducks were anesthetized with sodium pentobarbital, and liver tissues were collected for subsequent analyses.

### Cell isolation and cell culture

Liver tissues were isolated from 14-day-old duck embryos and rinsed in phosphate-buffered saline (PBS) before being finely minced on a culture dish. The fragmented tissue was then collected into a 50-mL centrifuge tube and repeatedly washed with PBS until the supernatant appeared transparent. Following PBS removal, type IV collagenase was introduced, and the tube was incubated at 37 °C for 15 min to allow enzymatic digestion. To halt digestion, DMEM supplemented with 10% FBS was added. The isolated hepatocytes were cultured in growth medium (GM) supplemented with insulin (0.57 mg/mL), penicillin-streptomycin (80 U/mL each), transferrin (5 mg/mL), dexamethasone (40 ng/mL), and L-glutamine (400 mg/mL). For model establishment, hepatocytes were exposed to increasing Cu^2+^ concentrations (0, 25, 50, 100, 200, 400 μmol/L). The IC₅₀ value for Cu^2+^ was calculated as 402.6 μmol/L, with 200 μmol/L selected as the optimal toxic dose. Similarly, cells were treated with a gradient of baicalin concentrations (0, 10, 20, 30, 40, 50 μmol/L), and 40 μmol/L was identified as the most effective concentration [36].

### CCK-8 assay

Primary duck hepatocytes were plated in 96-well culture plates (1 × 10^5^ cells/well in 100 μL medium) and allowed to adhere for 24 h. Following attachment, the cells were exposed to different doses of Cu or baicalin for four distinct treatment durations. Following the manufacturer’s protocol, 10 μL of CCK-8 (GLPBIO, USA) solution was added to each well, and the plates were incubated in a 5% CO₂ atmosphere at 37 °C for 1–4 h with light protection. Absorbance measurements were then taken using a microplate spectrophotometer to determine relative cell viability.

### Cell transfection

The si-LAPTM4B and its corresponding negative control siRNA (si-LAPTM4B-nc) were synthesized by Hanbio (Shanghai, China) at a final concentration of 100 nmol/L. Detailed oligonucleotide sequences are provided in Table S1. For transfection, RNAfit (Hanbio, China) was used as the delivery reagent. Following a 6-h incubation period, the transfection medium was removed and replaced with DMEM containing 2% fetal bovine serum. Subsequently, the transfected cells were exposed to 200 μmol/L Cu for further experimental procedures.

### The observation of ultrastructure

The ultrastructure of treated liver tissues and hepatocytes was analyzed by transmission electron microscopy (TEM) (Thermo Fisher, USA) as described previously [37].

### Histological examination

Tissue samples were processed following conventional protocols. Initially, fixation was carried out using paraformaldehyde, followed by dehydration in a graded ethanol series. After xylene-mediated dehydration, tissues underwent paraffin embedding. Histological evaluation was performed on H&E-stained sections using an Olympus light microscope (Japan) [38].

### Immunofluorescence staining

Liver tissues and hepatocytes were incubated with the corresponding antibodies (FAM134B, LC3B, LAMP1, LAPTM4B). Fluorescence intensity was detected using confocal fluorescence microscopy (Vutara352; Bruker, Germany) [39].

### Co-localization of endoplasmic reticulum with lysosomal probes

Cells were seeded into glass-bottomed confocal Petri dishes and cultured to the appropriate density. They were then incubated with ER-Tracker Red (Beyotime, Shanghai, China) and Lyso-Tracker (Beyotime, Shanghai, China) in medium for 20 min at 37 °C. The cells were washed twice with PBS and imaged by confocal microscopy (Olympus, Japan).

### Determination of oxidative stress indices

Commercial kits for measuring malondialdehyde (MDA), catalase (CAT), total superoxide dismutase (T-SOD), and hydrogen peroxide (H₂O₂) levels were provided by the Jiancheng Institute of Biological Engineering (Nanjing, China). All experiments were performed in strict accordance with the manufacturer’s instructions [40].

### Quantitative real-time PCR (RT-qPCR)

RNA isolation from both hepatic tissues and cultured hepatocytes was conducted with TriZol reagent (Vazyme Biotech, China) according to standard protocols. Subsequently, complementary DNA (cDNA) was synthesized employing a commercial reverse transcription system. RT-qPCR was carried out as established in prior methodology [41], using β-actin expression as the endogenous reference (relative fold change was calculated using the 2^−^^ΔΔCt^ method). The complete set of oligonucleotide primers utilized in this study is provided in Supplementary Table S2.

### Western blotting

Total proteins were extracted from 0.1 g of liver tissue and hepatocytes using RIPA lysis buffer (Vazyme Biotech, China). Protein concentrations were determined via a BCA assay kit (Solarbio, China), followed by standard Western blotting procedures. The following primary antibodies were used for detection: IRE1 (mouse, 1:800; Santa, USA), XBP1 (rabbit, 1:2,000; Wanleibio, China), FAM134B (rabbit, 1:5,000; Proteintech, China), TEX264 (rabbit, 1:2,000; Upinbio, China), LC3BI/II (rabbit, 1:1,000; Abmart, China), LAPTM4B (rabbit, 1:1,000; Proteintech, China), LAPTM5 (rabbit, 1:1,000; Wanleibio, China), LAMP1 (rabbit, 1:1,000; Wanleibio, China), LAMP2 (rabbit, 1:1,000; Wanleibio, China), and GAPDH (mouse, 1:5,0000; Proteintech, China). Band intensities were quantified using ImageJ software.

### Statistical analysis

Quantitative results are presented as mean ± standard deviation (SD) and were processed through GraphPad Prism 9 software (GraphPad Inc., USA) for statistical evaluation. Intergroup comparisons were conducted using one-way ANOVA with LSD post-hoc analysis. All experimental procedures were performed in triplicate, with a threshold of *P* < 0.05 considered statistically significant.

## Results

### Cu exposure induced liver injury and ER-phagy in ducks

**Fig. 1:**
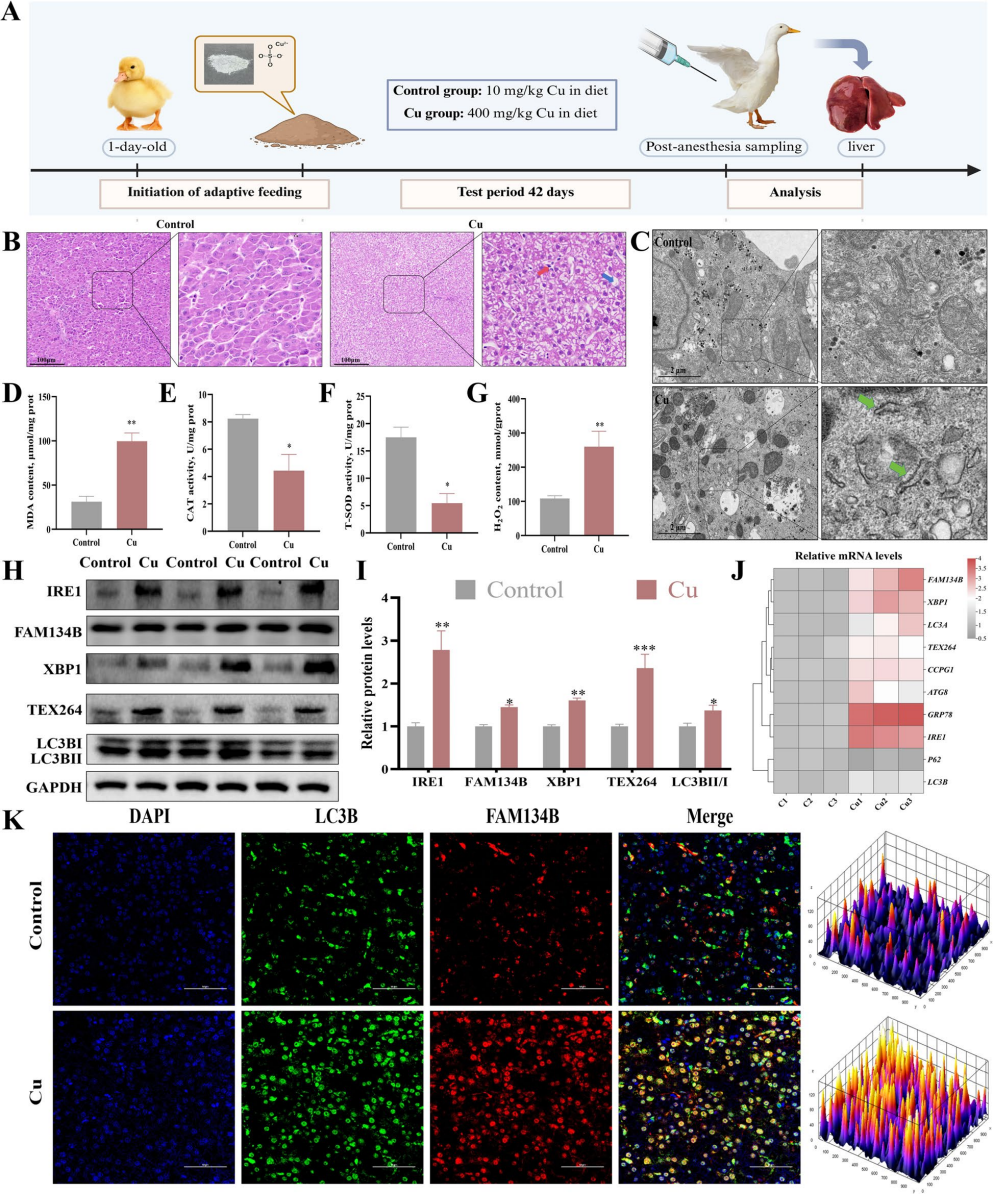
Cu exposure induced liver injury and ER-phagy in ducks. **A** Schematic of Cu exposure model. **B** Histopathological of the liver. Hepatocyte demonstrated vacuolation (blue arrow), inflammatory cell infiltration (red arrow). The scale bar is 100 μm. **C** Ultrastructural observations on the ER of the liver (green arrows). The scale bar is 2 μm. **D–G** The activities of CAT and T-SOD, and the content of MDA and H_2_O_2_. **H** and **I** The expression levels of proteins related to ER stress and autophagy. **J** The mRNA levels of genes associated with ER stress and autophagy. **K** Immunofluorescence observation of the LC3B and FAM134B co-localization. The scale bar is 50 μm. The data for each group were expressed as mean ± SD (*n* ≥ 3), ^*^P < 0.05, ^**^P < 0.01, ^***^P < 0.001

To investigate the effects of Cu exposure on the liver, we established an animal model of Cu exposure (Fig. 1A). Histopathological examination of liver tissue sections showed intact hepatocyte structures in the control group, with no significant pathological changes. In contrast, the Cu group exhibited marked hepatocyte vacuolization and inflammatory infiltration (Fig. 1B). Additionally, oxidative stress biomarkers—levels of MDA and H₂O₂—were significantly upregulated in the Cu group relative to the control, but the activities of CAT and T-SOD were notably downregulated (Fig. 1D–G). TEM analysis revealed considerable disruption of ER structures in Cu-exposed hepatocytes, with numerous autophagosomes present (Fig. 1C). We next assessed the mRNA and protein levels of ER stress and autophagy-related factors. It showed that Cu exposure significantly upregulated the mRNA levels of ER stress markers (*IRE1, XBP1, GRP78*) and autophagy-related genes (*FAM134B, CCPG1, TEX264, LC3A, LC3B, ATG8*), while downregulating the *P62* mRNA (Fig. 1J). Similarly, protein levels of IRE1, XBP1, FAM134B, TEX264, and LC3BII/I were significantly increased (Fig. 1H and I), further supporting the activation of both ER stress and autophagy pathways by Cu exposure. Immunofluorescence staining revealed a significant increase in the co-localization of FAM134B (an ER-phagy receptor) and LC3B (an autophagy marker protein) in the Cu group (Fig. 1K) further confirming the induction of ER-phagy by Cu exposure.

### Cu exposure led to lysosomal dysfunction in duck liver

**Fig. 2:**
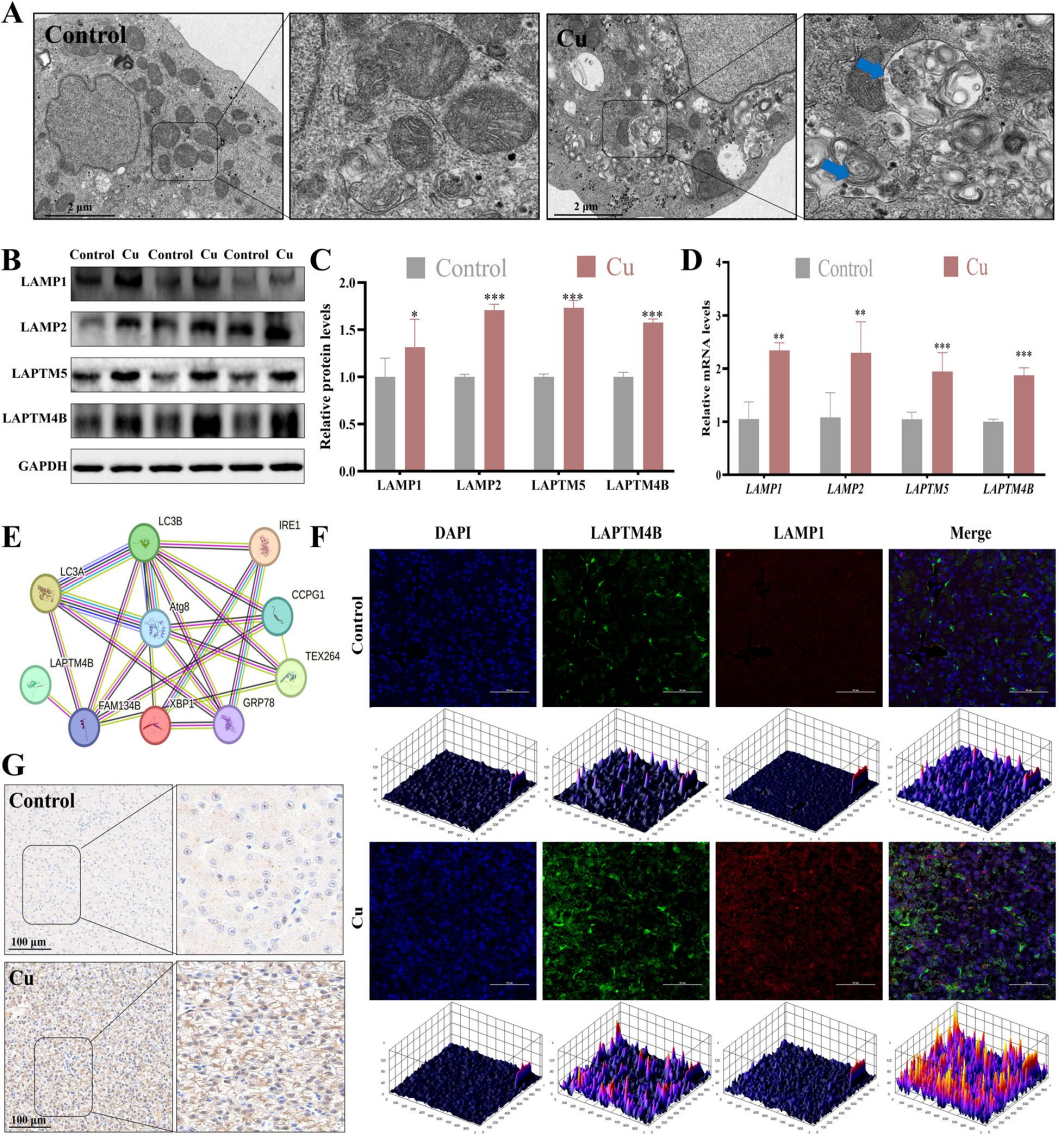
Cu exposure led to lysosomal dysfunction in duck liver. **A** Ultrastructural observations on liver autophagic lysosomes (blue arrows). The scale bar is 2 μm. **B** and **C** The expression levels of lysosome-related proteins. **D** The mRNA levels of lysosome-related genes. **E** PPI analysis of LAPTM4B with ER stress and autophagy proteins. **F** Immunofluorescence observation of the LAPTM4B and LAMP1 co-localization. The scale bar is 50 μm. **G** LAPTM4B immunohistochemical observation. The scale bar is 100 μm. ^*^P < 0.05, ^**^P < 0.01, ^***^P < 0.001

To further investigate the effects of Cu exposure on lysosomes, we observed the ultrastructure of liver tissues. The results revealed a significant increase in the number of autolysosomes in the Cu group (Fig. 2A). This finding, consistent with the previous observation of ER autophagy upregulation, led us to hypothesize that ER autophagy may be linked to lysosomal function. To test this hypothesis, we examined the expression of lysosomal membrane-associated factors. It showed that Cu exposure significantly upregulated the expression levels of LAPTM4B, LAPTM5, LAMP1, and LAMP2 (Fig. 2B–D). Further protein–protein interaction (PPI) analysis demonstrated that LAPTM4B interacts with autophagy-related molecules (Fig. 2E). Immunohistochemistry revealed that LAPTM4B expression level was significantly increased (Fig. 2G). Immunofluorescence staining revealed significant enhancement in the fluorescence signals of LAMP1 (a lysosomal marker) and LAPTM4B (a lysosome-associated protein) in the Cu group, along with increased co-localization (Fig. 2F). Collectively, these findings indicate that Cu exposure may result in lysosomal dysfunction.

### Cu exposure induced ER and lysosomal crosstalk disorder in duck hepatocytes

**Fig. 3:**
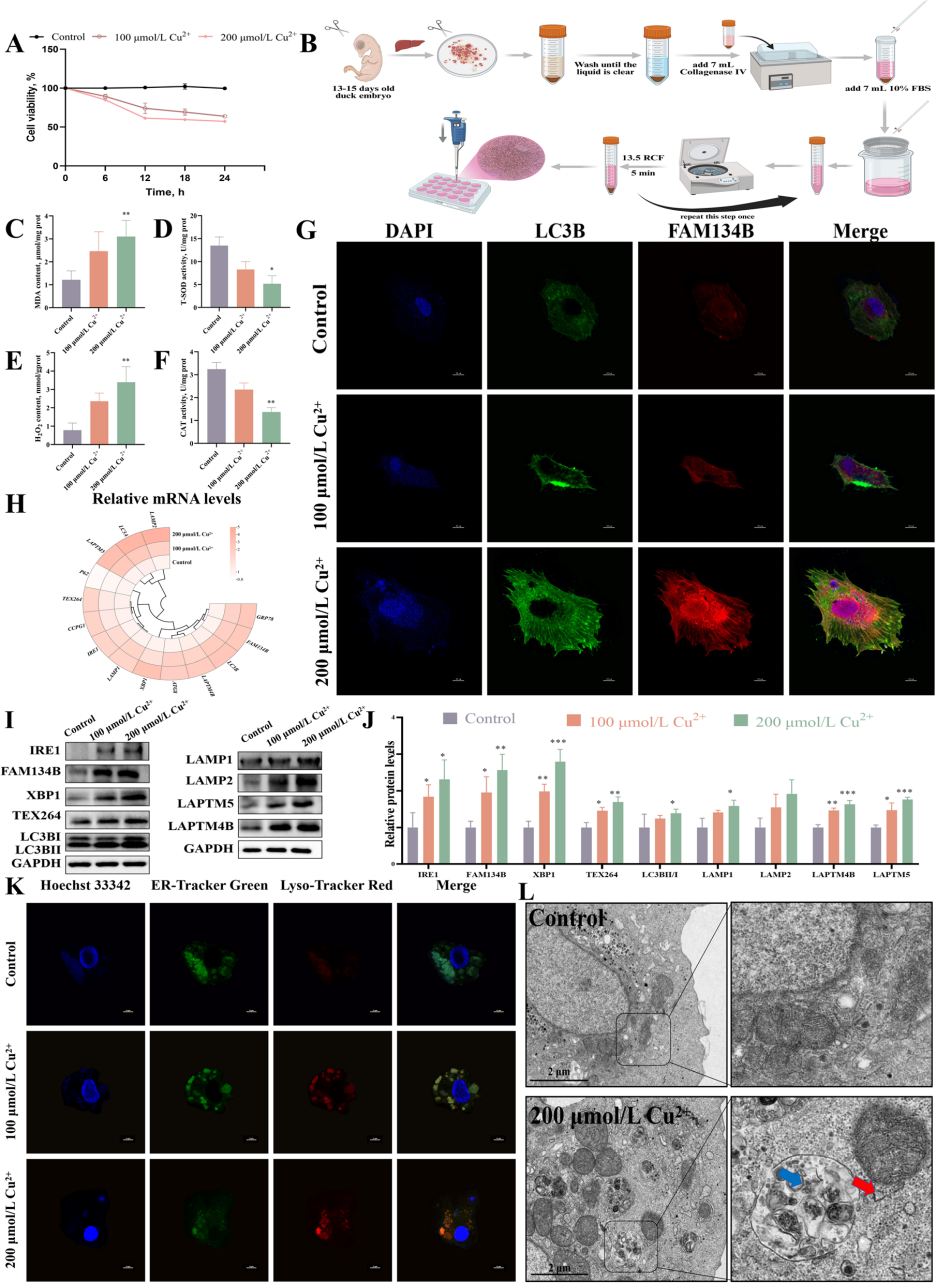
Cu exposure induced ER and lysosomal crosstalk disorder in duck hepatocytes. **A** Cell viability after 24 h of treatment with different concentrations of Cu^2+^. **B** Duck hepatocyte extraction process. **C–F** The activities of CAT and T-SOD, and the content of MDA and H_2_O_2_. **G** Immunofluorescence observation of the LC3B and FAM134B co-localization. The scale bar is 10 μm. **H** The mRNA levels of genes associated with ER-phagy and lysosomes. **I** and **J** The expression levels of proteins related to ER-phagy and lysosomes. **K** Immunofluorescence observation of the ER and lysosomes co-localization. The scale bar is 10 μm. **L** Ultrastructure of the hepatocytes. Swelling of the ER (red arrow) and the presence of numerous autolysosomes (blue arrow) were observed. The scale bar is 2 μm. ^*^P < 0.05, ^**^P < 0.01, ^***^P < 0.001 (compared with the control group)

To validate the in vivo results, we established a Cu exposure model using primary duck hepatocytes. Determined cell viability at various times and concentrations using the CCK-8 assay (Fig. 3A and B). Cu exposure significantly increased MDA and H₂O₂ levels, but reduced CAT and T-SOD activities (Fig. 3C–F). Immunofluorescence staining revealed significantly enhanced fluorescence signals of FAM134B and LC3B in the Cu-treated group (Fig. 3G). Cu exposure up-regulated the mRNA and protein expression levels of ER stress and autophagy-related genes and down-regulated the expression level of *P62* compared to control (Fig. 3H–J). Co-localization experiments with ER and lysosome probes revealed enhanced fluorescence signals in the Cu-treated group, indicating enhancement of interactions between the organelles and suggesting lysosomal damage (Fig. 3K). TEM observations showed significant ER swelling and an increased number of autolysosomes in hepatocytes of the 200 μmol/L Cu group (Fig. 3L). These results are consistent with the in vivo findings and further confirm the disruption of ER and lysosomal crosstalk by Cu exposure.

### Knockdown of LAPTM4B alleviated Cu-induced ER damage in duck hepatocytes

**Fig. 4:**
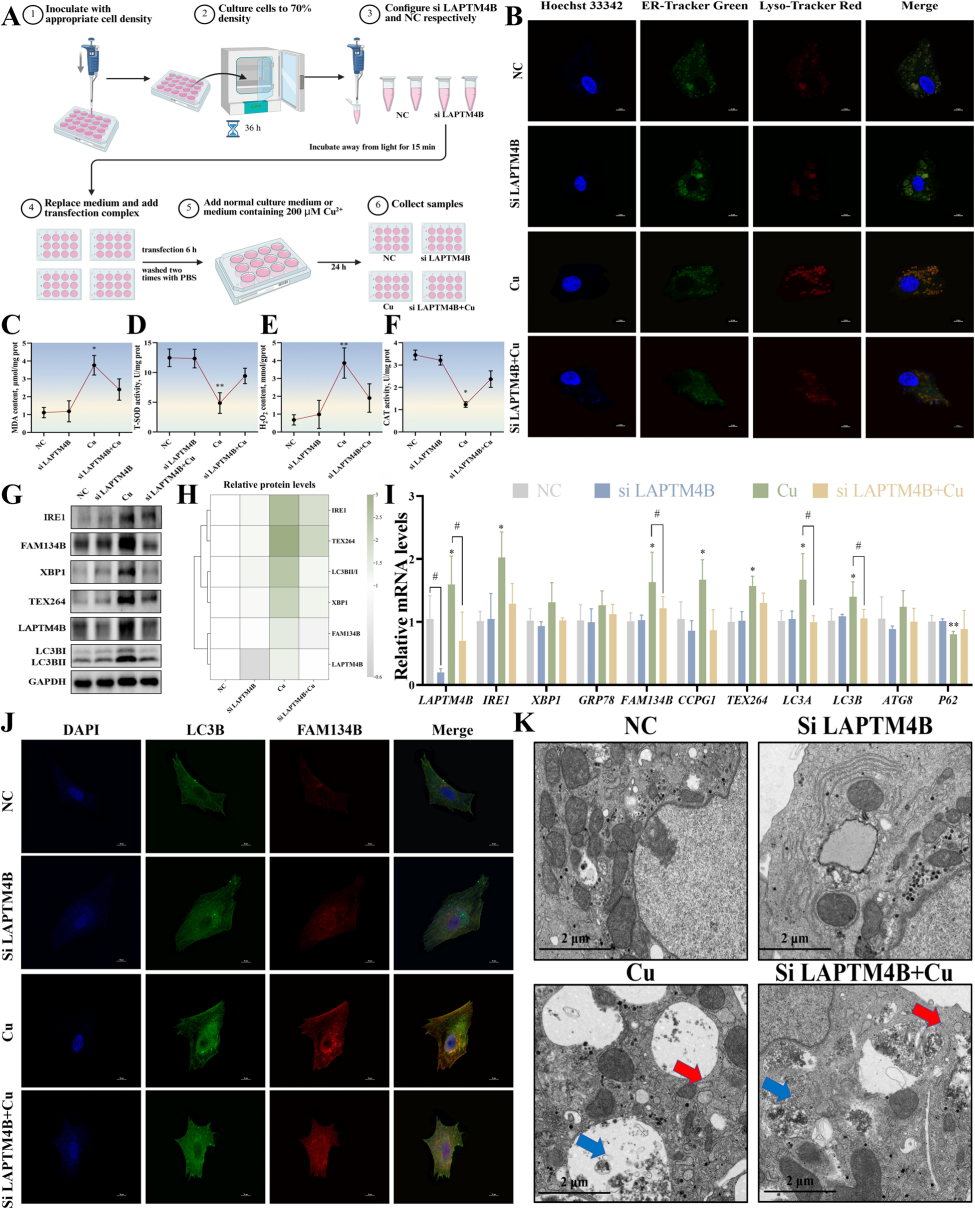
Knockdown of LAPTM4B alleviated Cu-induced ER damage in duck hepatocytes. **A** Cell group and treatment. **B** Immunofluorescence observation of the ER and lysosomes co-localization. The scale bar is 10 μm. **C–F** The activities of CAT and T-SOD, and the content of MDA and H_2_O_2_. **G** and **H** The expression levels of proteins related to ER-phagy and lysosomes. **I** The mRNA levels of genes associated with ER-phagy and lysosomes. **J** Immunofluorescence observation of the LC3B and FAM134B co-localization. The scale bar is 10 μm. **K** Ultrastructure of the hepatocytes. Swelling of the ER (red arrows) and the presence of numerous autolysosomes (blue arrows) were observed. The scale bar is 2 μm. ^*^P < 0.05, ^**^P < 0.01 (compared with the control group); ^#^*P* < 0.05

Building on the aforementioned findings that Cu exposure induced ER-phagy, we hypothesized that LAPTM4B could serve as a key upstream mediator of the interaction between lysosomes and autophagy receptors. As a key regulator of autophagy, LAPTM4B likely influences ER-phagy by promoting the fusion of lysosomes with autophagosomes. To test this hypothesis, we established a stable LAPTM4B knockdown model in duck hepatocytes using siRNA transfection (Fig. 4A). The efficient knockdown of LAPTM4B was confirmed by RT-qPCR and Western blot analysis. Co-localization experiments using fluorescent probes for the ER and lysosomes revealed that the fluorescence intensity of both organelles in the Cu-treated group was significantly reduced after LAPTM4B knockdown (Fig. 4B). This suggests that LAPTM4B knockdown mitigates abnormal interactions between the ER and lysosomes. Analysis of antioxidant indicators showed that LAPTM4B knockdown significantly reduced the levels of MDA and H₂O₂ in the Cu group, while increasing the activities of CAT and T-SOD (Fig. 4C–F). Cu exposure up-regulated the mRNA and protein expression levels of ER stress and autophagy-related genes and down-regulated the expression level of *P62* compared to controls. However, LAPTM4B knockdown significantly reversed the changes in the expression levels of these factors (Fig. 4G–I). Immunofluorescence staining showed significantly reduced fluorescence intensity of FAM134B and LC3B in the Cu-treated group after LAPTM4B knockdown (Fig. 4J). TEM observations demonstrated that LAPTM4B knockdown significantly mitigated the ER swelling and lysosomal damage caused by Cu exposure (Fig. 4K), highlighting the critical role of LAPTM4B in maintaining organelle structure and function.

### Ba targeted LAPTM4B to attenuate Cu-induced ER damage in duck hepatocytes

**Fig. 5:**
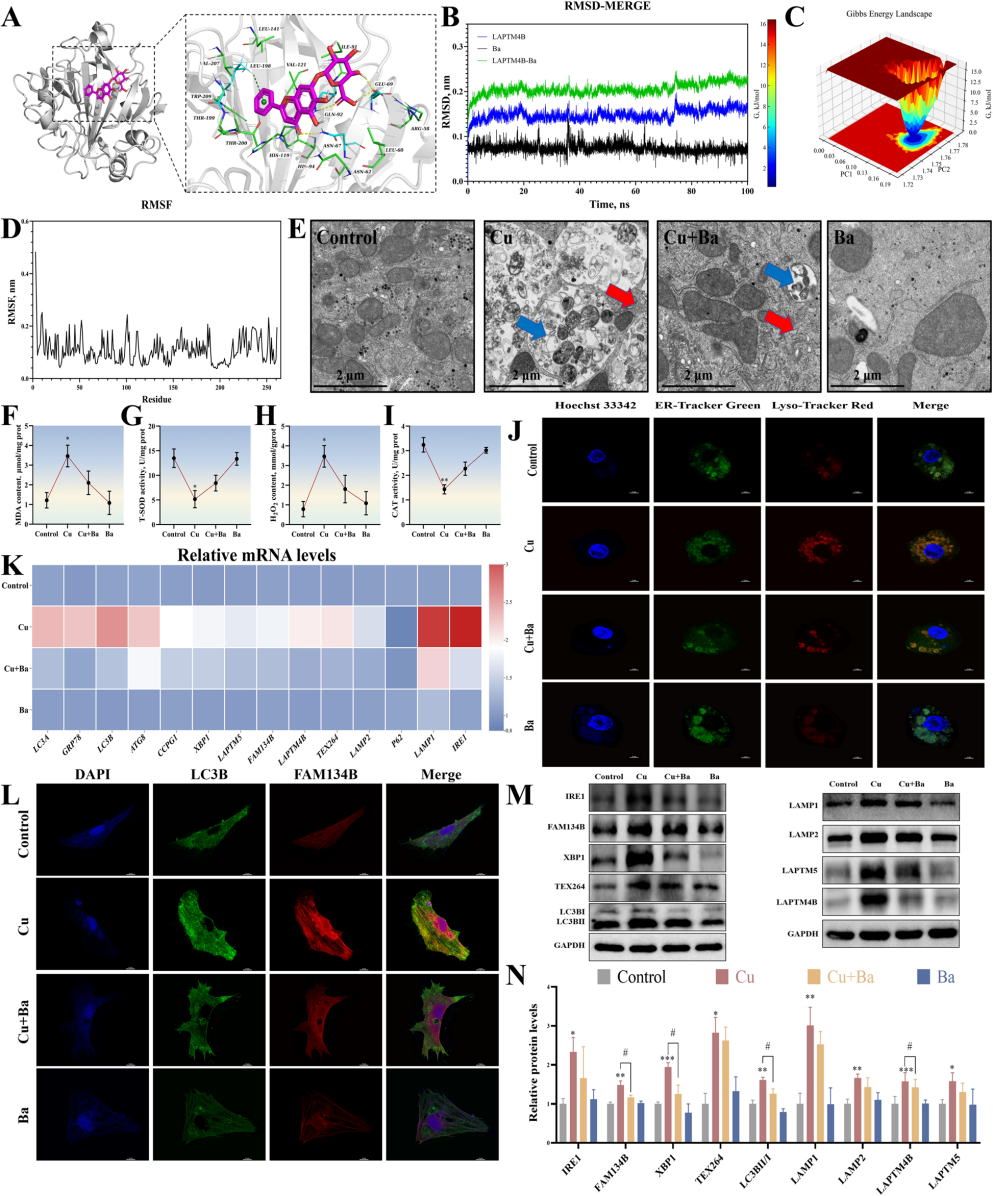
Ba targeted LAPTM4B to attenuate Cu-induced ER damage in duck hepatocytes. **A** Ba directly bound to LAPTM4B. **B–D** MD simulations were carried out to verify the molecular docking simulation results of LAPTM4B and Ba. **E** Ultrastructure of the hepatocytes. Swelling of the ER (red arrows) and the presence of numerous autolysosomes (blue arrows) were observed. The scale bar is 2 μm. **F–I** The activities of CAT and T-SOD, and the content of MDA and H_2_O_2_. **J** Immunofluorescence observation of the ER and lysosomes co-localization. The scale bar is 10 μm. **K** The mRNA levels of genes associated with ER-phagy and lysosomes. **L** Immunofluorescence observation of the LC3B and FAM134B co-localization. The scale bar is 10 μm. **M**–**N** The expression levels of proteins related to ER-phagy and lysosomes. ^*^P < 0.05, ^**^P < 0.01, ^***^P< 0.001 (compared with the control group); ^#^*P* < 0.05

To explore the potential mechanisms by which Ba alleviated Cu toxicity, we performed molecular docking (simulated using AutoDock and visualized with PyMOL) and molecular dynamics simulations (using GROMACS). The results showed that Ba exhibits strong binding affinity to LAPTM4B (Fig. 5A), and molecular dynamics simulations further confirmed the stability of this interaction (Fig. 5B–D). This suggests that Ba may exert its effects by directly targeting LAPTM4B. TEM observations revealed that Cu exposure caused severe damage to the structures of the ER and lysosomes, while the addition of Ba significantly alleviated these damages (Fig. 5E), indicating that Ba has a protective effect on organelles. Analysis of antioxidant indicators showed that Ba significantly reduced the levels of MDA and H₂O₂ in the Cu-treated group, while increasing the activities of CAT and T-SOD (Fig. 5F–I). Co-localization experiments using fluorescent probes for the ER and lysosomes demonstrated that Ba significantly reduced the fluorescence signal intensity of both organelles in the Cu group (Fig. 5J), suggesting that Ba mitigates abnormal interactions between the ER and lysosomes. Immunofluorescence staining revealed that the fluorescence signals of FAM134B and LC3B in the Cu group were significantly reduced after the addition of Ba (Fig. 5L). Cu exposure up-regulated the mRNA and protein expression levels of ER stress and autophagy-related genes and down-regulated the expression level of *P62* compared to controls. These changes were significantly reversed by Ba treatment (Fig. 5K, M, N). Furthermore, Ba also inhibited the expression level of LAPTM4B in the Cu group, suggesting that Ba may restore crosstalk with the ER by regulating LAPTM4B-mediated lysosomal function.

### Ba alleviated Cu-induced crosstalk disorder between ER and lysosomes in duck liver

**Fig. 6:**
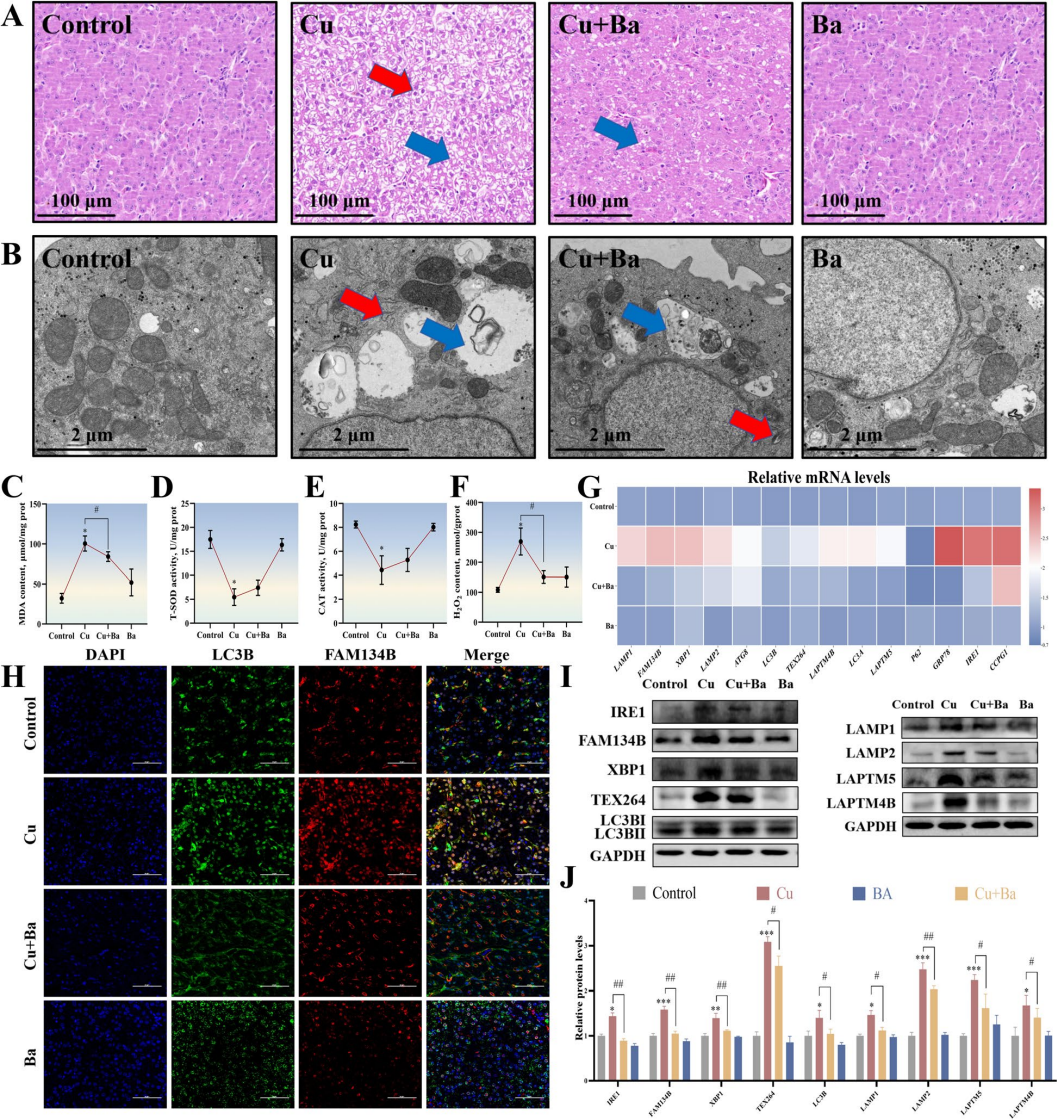
Ba alleviated Cu-induced crosstalk disorder between ER and lysosomes in duck liver. **A** Histopathological of the liver. Hepatocyte demonstrated vacuolation (blue arrows), inflammatory cell infiltration (red arrows). The scale bar is 100 μm. **B** Ultrastructure of the liver. Swelling of the ER was observed. (red arrows). The scale bar is 2 μm. **C**–**F** The activities of CAT and T-SOD, and the content of MDA and H_2_O_2_. **G** The mRNA levels of genes associated with ER stress and autophagy were measured. **H** Immunofluorescence observation of the LC3B and FAM134B co-localization. The scale bar is 50 μm. **I** and **J** The expression levels of proteins related to ER stress and autophagy. ^*^P < 0.05, ^**^P < 0.01, ^***^P < 0.001 (compared with the control group); ^#^*P* < 0.05, ^##^ *P*> < 0.01

Finally, to validate the in vitro findings, we conducted in vivo regression experiments. HE staining results showed significant vacuolization of hepatocytes in the Cu group, which was markedly alleviated after Ba treatment (Fig. 6A). TEM observations revealed severe damage to the ER and lysosomal structures in hepatocytes due to Cu exposure; however, these damages were significantly ameliorated upon Ba addition (Fig. 6B). Analysis of antioxidant indicators showed that Ba significantly attenuated the increase in MDA and H₂O₂ levels in the Cu group, while enhancing the activities of CAT and T-SOD (Fig. 6C–F). These results are consistent with the in vitro experiments. Immunofluorescence staining demonstrated that the fluorescence signals of FAM134B and LC3B in the Cu group were significantly reduced after Ba treatment (Fig. 6H). Similarly, mRNA and protein expression levels of autophagy and lysosomal membrane-associated proteins in the Cu group, which were reversed by Ba (Fig. 6G, I, J). The in vivo results were highly consistent with the in vitro findings, providing further evidence that Ba may alleviate Cu-induced abnormal crosstalk between hepatic endoplasmic reticulum and lysosomes by targeting LAPTM4B. These results support the potential application of Ba in the prevention and treatment of Cu-induced hepatotoxicity.

## Discussion

Cu, an essential trace element, regulates enzymatic activity and critical metabolic processes [42]. As a key component of cytochrome c oxidase (COX) in the mitochondrial respiratory chain, Cu enables electron transfer and ATP synthesis. Cu deficiency reduces COX activity by 30%−50%, impairing energy metabolism. Additionally, Cu supports iron metabolism via cuproproteins, catalyzing Fe^2+^ oxidation to Fe^3+^ for erythropoiesis [43]. However, Cu’s narrow safety window requires strict intake control: acute toxicity triggers oxidative gastrointestinal damage, hemolysis, and organ failure, while chronic overload causes hepatic/neurological Cu accumulation, manifesting as cirrhosis, neurological deficits, and Kayser-Fleischer rings [44]. Following oral ingestion, gastric acid converts Cu to Cu^2+^, absorbed via duodenal/jejunal CTR1/DMT1 transporters. Within enterocytes, Cu binds GSH/MT and is transported to blood via ATP7A. Albumin-bound Cu enters the liver through the portal vein, where hepatocytes uptake it via CTR1. ATOX1 directs Cu to target proteins, storage in Metallothionein (MT), or incorporation into Cu-dependent enzymes [45]. ATP7B exports Cu to ceruloplasmin for systemic use, with excess excreted via bile. This process is regulated by zinc and genetic factors (e.g., ATP7B deficiency), highlighting the liver’s central role in Cu homeostasis [46]. Although Cu-induced liver injury has been extensively studied [47], the mechanisms by which interactions between different organelles regulate this process remain unclear. This study reveals that Cu mediates the crosstalk between ER stress, autophagy, and lysosomal membrane proteins, providing new experimental evidence for the molecular mechanism of Cu-induced liver injury. The study elucidates the relationship between ER-phagy and lysosomal dysfunction under Cu exposure, builds a foundation for a theoretical framework of Cu-induced hepatotoxic effects, and fills a gap in the existing literature.

ER homeostasis plays a pivotal role in preserving cellular protein homeostasis, a process predominantly accomplished through the unfolded protein response (UPR), ER-mediated clearance of misfolded proteins, and coordinated interorganelle signaling [48]. Exposure to heavy metals has been shown to disrupt ER quality control systems, thereby inducing organelle dysfunction [49]. Early morphological studies demonstrated that Cu exposure caused ER lumen expansion and ribosome shedding in hepatocytes [50, 51]. In this study, TEM revealed increased ER swelling and elevated autophagosome numbers in Cu-exposed groups, indicating significant structural ER disruption and activation of quality control mechanisms. The ER quality control system employs autophagy to selectively degrade damaged components. ER-phagy, the core regulatory mechanism in this pathway, dynamically balances lysosomal degradation capacity [52]. Previous studies reported that Cu exposure upregulates ER stress markers (e.g., IRE1, XBP1) and autophagy-related proteins (e.g., LC3B), suggesting Cu interferes with ER homeostasis [53]. Here, we found that Cu not only activated ER stress pathways but also aberrantly upregulated key ER-phagy molecules (FAM134B, TEX264). Notably, Cu-induced ER-phagy exhibited distinct pathological features compared to physiological ER-phagy (e.g., as described by Chino et al. [54]): sustained FAM134B overexpression coincided with lysosomal dysfunction, trapping ER autophagy in a “high energy consumption–low efficiency” cycle. This pathology may arise from Cu-induced dual disruption of ER quality control, characterized by the induction of ER stress and hyperactivation of autophagy. Crucially, ER-phagy membrane remodeling relies on LC3B-FAM134B interaction, mediated by LC3B’s LIR (LC3-interacting region) and reticulon homology domains, which direct ER fragments to lysosomes [55]. Immunofluorescence analysis confirmed enhanced LC3B-FAM134B colocalization upon Cu exposure, further implicating aberrant ER-lysosomal crosstalk leading to ER-phagy activation in Cu-induced hepatotoxicity.

Lysosomes are not merely central to the cellular degradation system but are key players in ER quality control: the ER relies on lysosomal degradation to clear out damaged components via autophagy [56, 57]. Recent studies have transcended the conventional cognitive framework, revealing that lysosome function both as the terminal station of material degradation and as a nexus for signal integration, actively contributing to metabolic reprogramming and stress adaptation through molecular mechanisms such as the mTORC1-TFEB axis regulatory network and calcium signal transduction. In the context of heavy metal toxicity research, the specific interference of Cu ions on lysosomal function has garnered increasing attention [58–60]. Recent evidence suggests that Cu can disrupt membrane integrity by inducing oxidative modification of lysosomal membrane proteins (e.g., LAMP-family proteins), or interfere with lysosomal acidification by inhibiting the function of the V-ATPase proton pump, which can lead to blockage of autophagy flow [61]. However, the specific manner in which Cu-induced hepatotoxicity disrupts the lysosome-ER synergy network remains to be elucidated. This study elucidates the molecular underpinnings of Cu-induced lysosomal dysfunction, manifesting in aberrant expression of lysosomal membrane-associated molecules and an accumulation of defective autophagic lysosomes. A mechanistic analysis centered on the lysosomal membrane-associated protein family (LAMP1/2, LAPTM4B/5, which are pivotal for maintaining lysosomal membrane homeostasis, revealed that LAPTM4B possesses a distinct autophagy regulation function [62, 63]. The results of the protein–protein interaction (PPI) validation suggest that LAPTM4B interacts directly with the ER-phagy receptor FAM134B, which indicates the possibility of its involvement in ER-lysosomal crosstalk regulation. Construction of a LAPTM4B knockdown (KD) cell model reveals that KD treatment not only reverses Cu exposure-induced oxidative stress, ER stress, autophagy, and lysosomal dysfunction but also suppresses abnormal ER-lysosome co-localization triggered by Cu, suggesting its potential therapeutic application in diseases involving ER-lysosomal crosstalk. ER-lysosomal crosstalk disorder has been shown to promote premature docking of the ER membrane to the lysosome, resulting in the “forced wrapping” of improperly folded ER fragments to form functionally defective autophagic lysosomes that are unable to be efficiently degraded [64, 65]. This aberrant interaction disrupts the regulatory balance of ER proteins, leading to a positive feedback loop that exacerbates lysosomal dysfunction. This finding suggests that targeted regulation of the dynamic balance at the lysosome-ER interface may be a new strategy to intervene in Cu-induced hepatotoxicity.

Natural compounds have demonstrated unique advantages in the treatment of metal toxicity due to their multi-target regulation. For instance, Ba has been shown to mitigate heavy metal-induced liver injury by chelating free Cu ions and suppressing ROS generation, as evidenced by earlier studies [66, 67]. Studies have demonstrated that Ba may enhance cellular antioxidant defense by activating the Nrf2/ARE signaling pathway, offering new insights into its detoxification mechanism [68, 69]. Furthermore, evidence suggests that Ba can ameliorate neuroinflammation through the PI3K/AKT/FoxO1 pathway, highlighting its multi-pathway synergistic effects [70, 71]. However, the ability of these compounds to repair lysosomal-ER axis imbalance remains to be elucidated. Molecular docking simulations revealed that the molecular conformation of Ba can form a dense hydrogen bonding network with the intracellular structural domain of LAPTM4B. Furthermore, molecular dynamics simulations revealed that the Ba-LAPTM4B complex exhibited consistent low RMSD fluctuations (< 2 A) within a phospholipid bilayer environment for up to 200 ns, thereby confirming the stability of the complex under physiological conditions. In light of these findings, the present study centered on the recently identified LAPTM4B-mediated ER-lysosomal axis imbalance mechanism, with the objective of systematically investigating the potential of Ba to reverse Cu toxicity by regulating this pathway. In the present study, we found that Ba significantly reversed the ER-lysosome disorder triggered by Cu exposure by targeting LAPTM4B. This was evidenced by the expression of ER damage markers, such as IRE1 and FAM134B, and lysosomal functional proteins, such as LAMP1, were regulated. The available evidence suggests that the protective effect of Ba against Cu toxicity may stem from multiple mechanisms synergistically: direct metal chelation, Nrf2-mediated antioxidant defense, and remodeling of the ER-lysosomal interaction network through the modulation of LAPTM4B. This multi-targeted intervention property renders it more promising for therapeutic applications in complex pathological environments.

## Conclusion

Collectively, Cu exposure disrupts hepatic ER-lysosomal crosstalk, leading to ER lysosomal damage, which can be mitigated by Ba through selective targeting of LAPTM4B, ultimately alleviating Cu-induced hepatotoxicity.

## Supplementary Information

Supplementary Material 1: Table S1 Primer sequences for target genes. Table S2 Primer sequences for target genes.

## Data Availability

The datasets generated and/or analysed during the current study are not publicly available due [REASON WHY DATA ARE NOT PUBLIC] but are available from the corresponding author on reasonable request.

## Abbreviations

Ba: Baicalin
Cu: Copper
CAT: Catalase
ER-phagy: Endoplasmic reticulum autophagy
ER: Endoplasmic reticulum
H₂O₂: Hydrogen peroxide
MDA: Malondialdehyde
ROS: Reactive oxygen species
T-SOD: Total superoxide dismutase
