# Supplementary material for: LAPTM4B as a key regulator in the copper-induced endoplasmic reticulum–lysosome interplay disorder in duck liver and the protective role of baicalin
Source: J Anim Sci Biotechnol. 2025 Sep 1;16:121. doi: 10.1186/s40104-025-01255-0 (PMC12400699; doi:10.1186/s40104-025-01255-0)
Supplement: Supplementary file 1 — Supplementary Material 1: Table S1 Primer sequences for target genes. Table S2 Primer sequences for target genes. [file 40104_2025_1255_MOESM1_ESM.docx]

Table S1

Primer sequences for target genes

| Name | Sense Strand | Antisense Strand |
| --- | --- | --- |
| CY3 siRNA NC | UUCUCCGAACGUGUCACGU TT | ACGUGACACGUUCGGAGAA TT |
| siRNA NC | UUCUCCGAACGUGUCACGU TT | ACGUGACACGUUCGGAGAA TT |
| si LAPTM4B | CCAGCACAAUUCAGGACUATT | UAGUCCUGAAUUGUGCUGGTT |

Table S2

Primer sequences for target genes

| Gene | 5′-Primer (F) | 3′-Primer (R) |
| --- | --- | --- |
| *β-actin* | GATGGCTCCGGTATGTGCAA | CAACCATCACACCCTGATGTC |
| *IRE1* | GCGGACAGGAGCAATCAAATG | CATCTGAACTGCGACACGGA |
| *XBP1* | GGGCAACCAAACCAATATGCT | GCAAGGCTACAAGGAGAGGG |
| *GRP78* | CTGCTTGACGTGTGTCCTCT | TCACAGTTGGCTGGTTGTCA |
| *LAPTM4B* | AGGATGGAGAACGTGGCATT | TGAAGGTCGAAGTGGGCATC |
| *FAM134B* | TCTGACCCCTTGGAGAGTGT | TCTTGCCTGGAACTGATGACT |
| *CCPG1* | TGGTGAAGAAGCTCGCAGTT | CCTCATTGCCCCATTCCCTT |
| *TEX264* | TGTCTACTCGGGGCTCTTCA | CTGCCCCGATTCACCATAGG |
| *LC3A* | TCCTTGTCCCAGACCATGTC | GCCATCCTCATCCTTCTCCT |
| *LC3B* | ACAGTACAGACGAGCACCTC | CCAGAAAACTGTCACACGCA |
| *ATG8* | AATCCGAAAGCGAATCCACCT | TGCCATAGACGCTCTCATCAC |
| *P62* | TGGGTTTGGTAGCTCTGCAC | GCCAATGTTGAAGCCGGTTG |
| *LAPTM4B* | AGGATGGAGAACGTGGCATT | TGAAGGTCGAAGTGGGCATC |
| *LAPTM5* | TTCAACCTGCTCCTTGGTGT | AGCCAGGAATGGGATCTTCG |
| *LAMP1* | GACAACACGTCTGCCTTCCT | GCTCTCAGTTCGCTCATGCT |
| *LAMP2* | TGGGTTCAACCATTCCTCGT | AAATTGCTCGTATCCGGCAT |
